# Can biological control involving predatory mites mitigate plant stress caused by phytophagous mites?

**DOI:** 10.1007/s00425-026-05004-z

**Published:** 2026-04-29

**Authors:** Wesley Borges Wurlitzer, Julia Renata Schneider, Mateusz Labudda, Julia Huppes Majolo, Marcelo Lattarulo Campos, Joaquim A. G. Silveira, Daniel Guimarães Silva Paulo, Maria Goreti de Almeida Oliveira, Noeli Juarez Ferla

**Affiliations:** 1 Laboratory of Acarology, Tecnovates, Universidade do Vale do Taquari, Univates, Lajeado, Rio Grande Do Sul Brazil; 2 Postgraduate Program in Biotechnology, Universidade do Vale do Taquari, Univates, Lajeado, Rio Grande Do Sul Brazil; 3 BIOAGRO‑Instituto de Biotecnologia Aplicada à Agropecuária /INCT‑Interações Planta‑Praga, Universidade Federal de Viçosa, UFV, Viçosa, Minas Gerais Brazil; 4 Department of Biochemistry and Microbiology, Institute of Biology, Warsaw, University of Life Sciences-SGGW, Nowoursynowska 159, 02-776 Warsaw, Poland; 5 Integrative Plant Research Laboratory, Departamento de Botânica e Ecologia, Instituto de Biociências, Universidade Federal de Mato Grosso, UFMT, Cuiabá, Mato Grosso Brazil; 6 Plant Metabolism Laboratory (LabPlant), Universidade Federal do Ceara, UFC, Fortaleza, Ceará Brazil; 7 Department of Entomology, Universidade Federal de Viçosa, Viçosa, Minas Gerais Brazil; 8 Postgraduate Program in Environment and Development, Universidade do Vale do Taquari, Univates, Lajeado, Rio Grande do Sul Brazil

**Keywords:** Oxidative stress, Pest management, Plant physiology, Plant stress, Herbivory, Stress mitigation

## Abstract

**Main conclusion:**

**The biological control of phytophagous mites mediated by predatory mites promotes the maintenance of plant physiology by mitigating damage and preserving key traits related to photosynthesis and development.**

**Abstract:**

The immune system of plants, upon perceiving the presence of phytophagous mites, triggers signals mediated by reactive oxygen species (ROS). Under intense infestations, these redox signals can trigger oxidative stress, which compromises vital characteristics related to plant fitness. The use of pesticides as a management strategy is increasingly limited by resistance and collateral physiological impacts on plants. The release of predatory mites has emerged as an effective and sustainable biological approach. Predator-mediated foraging may suppress phytophagous mite infestations and mitigate the physiological stress of plants by limiting metabolic expenditures and physiological disturbances related to photosynthesis, growth, and reproduction. Thus, we review the benefits and risks of signals mediated by ROS in plants under attack by phytophagous mites. We conceptualize a stress state for plants under attack by these organisms and describe the benefits of foraging predatory mites reported in the literature from a meta-analytical perspective. Based on existing studies, we show that these natural enemies mitigate damage and the intensification of foliar chlorosis, limiting impacts on leaf area, the number of leaves, and the size of the plants. Furthermore, we speculate how the intensity of stress in the plant could act as a key point in the signals emitted to attract predatory mites. Finally, we emphasize the urgency of integrating this new perspective into future studies to improve the evaluation of the efficiency of natural enemies to benefit plant performance.

**Supplementary Information:**

The online version contains supplementary material available at 10.1007/s00425-026-05004-z.

## Introduction

Phytophagous mites, particularly those belonging to the Tetranychidae family, infest more than 1000 plant species, many of which are of agricultural relevance (Moraes et al. 2024). The use of pesticides has limited effectiveness because of the recurring development of resistance in these organisms (Moraes et al. 2024). In addition, undesirable xenobiotic effects are induced in the host plant, leading to potentially lethal oxidative disorders (Macar 2021; Hatamleh et al. 2022). As a sustainable and beneficial strategy, classical or augmentative biological control mediated by predatory mites is effective (Fontes and Valadares-Inglis 2020; Moraes et al. 2024). For example, the release of the predatory mite *Amblyseius herbicolus* (Chant) (Phytoseiidae) on pepper plants (*Capsicum frutescens* L.) efficiently controlled the infestation by the broad mite *Polyphagotarsonemus latus* (Banks) (Tarsonemidae), mitigating the intensification of leaf damage and plant mortality, while preventing reductions in fruit weight (Rodríguez-Cruz et al. 2017). Thus, the importance of these natural enemies goes beyond biological control, as they also maintain essential physiological characteristics in plants (Argolo et al. 2020; Pijnakker et al. 2022; Wang et al. 2025).

Plants themselves possess defense mechanisms that act to reduce herbivore feeding activity. Induced defenses, for example, are triggered immediately upon perception of the herbivore and intensify in response to tissue damage, dependent on the feeding behavior of the herbivore species (Wurlitzer et al. 2024a). During this process, phytophagous mites secrete effectors to modulate plant perception and hinder the activation of the plant immune system (Snoeck et al. 2022). However, various molecular cues, such as herbivore-associated molecular patterns (HAMPs) and damage-associated molecular patterns (DAMPs), act as warning signals for plant cells (Couto and Zipfel 2016; Reymond 2021; Snoeck et al. 2022; Cui et al. 2024; Wurlitzer et al. 2024a). These elicitors are recognized by the pattern recognition receptors (PRRs) (Snoeck et al. 2022), triggering endogenous signals, such as calcium ion (Ca^2+^) influxes and reactive oxygen species (ROS) production, which culminate in the activation of multiple hormonal pathways and defense-related gene expression (Campos et al. 2014; Snoeck et al. 2022).

Under intense infestation, excessive ROS accumulation can become detrimental, causing physiological disturbances in plant cells (Mittler 2017; Gogoi et al. 2024; Wurlitzer et al. 2024b). In addition to the lesions caused by herbivory itself, the excessive accumulation of ROS triggers early oxidative disorders that compromise key developmental processes, leading to physiological stress in the plant (Silveira and Sousa 2024; Wurlitzer et al. 2024b). Although the evidence in the literature is limited, it is likely that plants under attack by phytophagous mites reduce their physiological development in order to invest in oxidative defenses in an attempt to maintain ROS waves for essential signaling processes. This physiological strategy is conceptually supported under scenarios involving abiotic disturbances (Ferreira-Silva et al. 2011; Lima et al. 2018). For example, cashew plants under water deficit exhibit reduced growth as an adaptive physiological strategy to invest in oxidative protection, contributing to the maintenance of photosynthetic activity (Lima et al. 2018).

Furthermore, when under attack by phytophagous mites, plants also activate induced defense pathways involved in plant immunity to reduce the performance of the herbivores (Niinemets et al. 2013; Pearse et al. 2020; Buffon et al. 2021; Wurlitzer et al. 2024a). Similar to oxidative protection, herbivory forces plants to balance growth and defense, often downregulating genes involved in growth and photosynthesis to prioritize the synthesis of anti-digestive proteins and secondary metabolites (Campos et al. 2014; Karasov et al. 2017; Sestari and Campos 2022; Ku et al. 2024). In the case of phytophagous mites, plant-induced defenses, such as the synthesis of protease inhibitors (PIs), not only cause herbivore mortality through the inhibition of gut proteolytic activity but also indirectly contribute to mitigating oxidative disturbances caused in the plant itself (Arnaiz et al. 2018). Considering that these induced defenses are not always sufficient to control herbivory, a state of physiological stress can be reached (Niinemets et al. 2013).

Plant stress depends on the plant’s ability to tolerate a given stressor. It may be reversible if it remains within physiological limits or irreversible if these limits are exceeded (Lichtenthaler et al. 1996; Mosa et al. 2017). Silveira and Sousa (2024) proposed a systemic concept for diagnosing plant stress, integrating early metabolic defense responses and oxidative disturbances with the late effects on plant performance (Züst and Agrawal 2017). Applying this proposal to the management of phytophagous mites, commonly used pesticides may intensify the oxidative disturbances caused by the infestation, increasing the metabolic defense reprogramming and negatively impacting the development of host plants (Macar 2021; Hatamleh et al. 2022; Moraes et al. 2024). In contrast, the suppression of these mites with natural enemies may act in the opposite direction (Argolo et al. 2020; Pijnakker et al. 2022), suggesting that suppressing phytophagous mites with predatory mites may mitigate the stressful physiological state in the host plant.

From this perspective, considering that pesticides can intensify the physiological disturbances caused by phytophagous mites, while predatory mites suppress infestations without inducing additional disturbances (Fontes and Valadares-Inglis 2020), this review highlights the potential of these natural enemies in mitigating physiological stress. First, we describe the mechanisms of perception of phytophagous mite attack in plants, highlighting the central role of ROS in immune signaling. We conceptualize how the intensification of these signals is closely related to a state of stress in plants under attack by phytophagous mites and then characterize how the suppression of phytophagous mites by the foraging of predatory mites can mitigate physiological plant stress. To contextualize, we gather evidence from a meta-analytical perspective, considering aspects related to plant fitness, and we propose two hypothetical mechanisms that plants may adopt to attract these natural enemies to mitigate the stress associated with infestation at individual and agroecosystem levels. Finally, we conclude and discuss future directions for this emerging topic.

## The central role of ROS in plant immunity

During herbivory, endogenous cell fragments released from the damaged plant cells can act as DAMPs, which are perceived by specific PRRs. These PRRs are primarily classified into two main types of receptor kinases (RKs): (*i*) those that recognize peptide elicitors through receptors such as PeP receptor (PEPR) and systemin receptor (SYR), and (*ii*) those that detect signaling purines, mediated by lectin receptor kinase (LecRK) (Ngou et al. 2022; Snoeck et al. 2022). In the case of phytophagous mites, particularly tetranychids (spider mites), feeding occurs through the piercing and sucking of mesophyll cell contents using stylets (Wurlitzer et al. 2024a). Evidence from *Arabidopsis thaliana* plants infested with *Tetranychus urticae* Koch (Tetranychidae) shows the induction of genes related to DAMP signaling, including PEPR1, PEPR2, LecRK4.1, and LecRK1.9 (Zhurov et al. 2014). These genes also contribute to signaling cascades involving the production of hydrogen peroxide (H_2_O_2_), a hallmark of early defense activation (Zhurov et al. 2014).

**Fig. 1:**
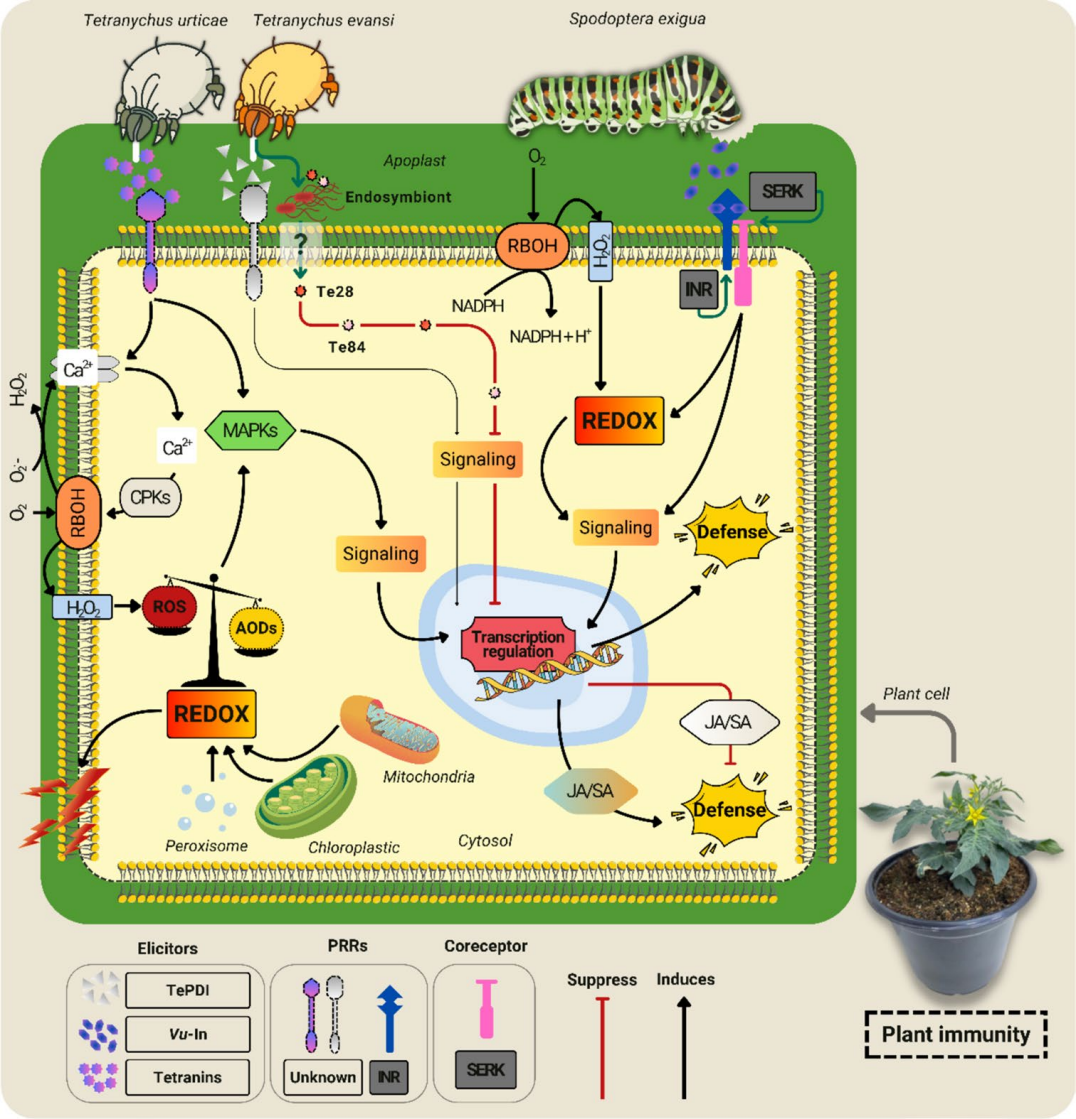
ROS signaling as an integral part of plant immunity. Elicitors present in the oral secretions of the caterpillar *Spodoptera exigua* are recognized by PRRs, such as INR, and their co-PRR SERK, triggering downstream defense responses that depend on redox signals. Similar mechanisms occur in the interaction with phytophagous mites. The tetranins secreted by *Tetranychus urticae* lead to the opening of ion channels, influx of Ca^2^⁺, and activation of CPKs, which regulate RBOH production of O_2_^−^ and H_2_O_2_ in the apoplast. H_2_O_2_ crosses the plasmalemma via peroxyporins to the cytosol. The fine-tuning of these ROS through the AODs leads to influxes of signals that participate in the activation of MAPKs. The MAPKs initiate transcriptional reprogramming, leading to the activation of genes involved in JA, SA, and ET, and the induction of defense compounds. In cases of intense disturbances, the disruption of redox homeostasis leads to lipid peroxidation (indicated by a lightning bolt symbol). *Tetranychus evansi* also triggers signaling events by ROS. The main key feature of this mite involves the secretion of effector proteins and endosymbionts capable of suppressing defense responses. The mechanism of endosymbiont entry is still unknown. The proportions of the graphic elements are illustrative only, without taking into account actual dimensions. Abbreviations: *PRRs* pattern recognition receptors, *INR*, inceptin receptor, *SERK*, somatic embryogenesis receptor kinase, *CPKs*, calcium-dependent protein kinases, *RBOH*, respiratory burst oxidase homolog, *O*_*2*_^*−*^ superoxide, *H*_*2*_*O*_*2*_ hydrogen peroxide, *V*_*m*_ membrane potential, *ROS* reactive oxygen species, *AODs* antioxidants, *MAPKs* mitogen-activated protein kinases, *JA* jasmonic acid, *SA* salicylic acid, *ET* ethylene, *REDOX* redox homeostasis, *Te28 and Te84* effector proteins. The thickness of the arrow indicates the intensity of the event. Plant represented by *Solanum lycopersicum*

Regarding PRRs involved in the recognition of HAMPs, these receptors are fewer in number than those that recognize DAMPs and often require co-receptors to activate effective signaling and defense responses (Ngou et al. 2022; Snoeck et al. 2022). The ability of most PRRs to directly recognize herbivore-derived ligands is not yet fully established (Ngou et al. 2022). One of the best-characterized examples involves the herbivore caterpillar *Spodoptera exigua* (Hübner) (Noctuidae) feeding on cowpea beans [*Vigna unguiculata* (L.) Walp]. In this interaction, herbivory induces the release of the HAMP inceptin *Vu-*In, a peptide derivative from the proteolysis of the chloroplastic enzyme ATP synthase. When the caterpillar regurgitates digestive fluids or oral secretions back onto the wounded leaf surface, *Vu*-In is deposited on the plant’s tissues, where it acts as a signal that herbivory is occurring. The recognition of *Vu*-In occurs through the inceptin receptor (INR), which is specific to legumes and functions in association with leucine-rich repeat receptor-like kinases (LRR-RLKs), such as somatic embryogenesis receptor kinase (SERK), to activate ROS-mediated defense pathways (Fig. 1) (Steinbrenner et al. 2020).

Regarding phytophagous mite elicitors, the systematic review of Snoeck et al. (2022) suggests that most of the available evidence refers to partial or indirect aspects related to PRRs. Only recently have specific salivary proteins been identified as potential elicitors. In *Tetranychus evansi* Baker & Pritchard (Tetranychidae), the proteins Te16 and TePDI (protein disulfide isomerase) were shown to induce an oxidative burst in tobacco (*Nicotiana benthamiana* Domin) (Cui et al. 2023, 2024). Although PRRs involved in the perception of TePDI have not yet been characterized, the accumulation of H_2_O_2_ triggered by Te16 appears to be mediated by a co-PRR, the LRR-RLK-like BRI1-associated kinase 1 (BAK1) (Cui et al. 2023, 2024). At the same time, the accumulation of H_2_O_2_ is unleashed by TePDI, other effector proteins, such as Te28 and Te84, are likely to attenuate this oxidative signal (Cui et al. 2023). *Tetranychus evansi* also harbors several endosymbionts in its digestive tract, which can suppress the activity of enzymes responsible for activating key hormonal pathways involved in the activation of plant defenses (Ribeiro et al. 2020, 2022). Investigating how these symbionts influence ROS fluxes by specific mite elicitors could provide essential insights into the modulation of plant defense signaling.

Recent studies have also characterized several elicitors related to the recognition of and defense against *T. urticae* (Ojeda-Martinez et al. 2021; Endo et al. 2025). For example, egg-associated molecular pattern (EAMP) candidates triggered influxes of cytosolic Ca^2^⁺ and ROS in Arabidopsis (Ojeda-Martinez et al. 2021). Similarly, HAMP Tetranins 1–4 (Tet1, Tet2, Tet3, and Tet4) elicited distinct signaling responses across different species. In cucumber leaves, Tet1, Tet2, and Tet3 triggered influxes of Ca^2^⁺, while the accumulation of ROS was mediated by Tet1, Tet2, and Tet4 (Endo et al. 2025). In bean plants (*Phaseolus vulgaris* L.), only Tet2 and Tet4 induced ROS accumulation (Iida et al. 2019; Endo et al. 2025). Considering that the expression of Tet3 and Tet4, as well as the influx of Ca^2^⁺ and ROS accumulation, was higher in beans than in cucumber (Endo et al. 2025), *T. urticae* may adjust the secretion intensity of the HAMPs according to the metabolic robustness of each host plant species. Indeed, this scenario suggests that ROS signaling is a key and frequently activated response in plants infested by phytophagous mites.

ROS production in plants under herbivory results from a complex and tightly regulated network of cellular processes (Gogoi et al. 2024). In addition to immune-mediated binding of elicitors to their PRRs, ROS may be generated by respiratory burst oxidase homolog (RBOHs) proteins, which reduce atmospheric oxygen (O_2_) to O_2_^−^ in the apoplast. This radical is then rapidly dismuted into H_2_O_2_ by the enzyme superoxide dismutase (SOD) (Gogoi et al. 2024). The resulting H_2_O_2_ readily crosses the plasma membrane via peroxyporin-type aquaporins (Mittler 2017), reaching the cytosol, where it accumulates along with ROS continuously generated in chloroplasts, mitochondria, and peroxisomes, which are also sources of other ROS, such as singlet oxygen (^1^O_2_). In parallel, plants maintain optimal ROS levels through the action of antioxidant defenses, including ascorbate peroxidase (APX), catalase (CAT), glutathione reductase (GR), and proline (Peláez-Vico et al. 2024). In this scenario, ROS, particularly H_2_O_2_, function as redox messengers (Peláez-Vico et al. 2024), triggering essential signaling cascades without causing oxidative damage (Yuan et al. 2021; Gogoi et al. 2024).

In plant responses to phytophagous mites, H_2_O_2_ may mediate two defense pathways (Schweighofer et al. 2007; Peláez-Vico et al. 2024; Wurlitzer et al. 2024a, 2025). The first likely involves the oxidation of specific cysteine residues in target proteins, leading to the activation of mitogen-activated protein kinases (MAPKs) and specialized transcription factors (Schweighofer et al. 2007). Once activated, MAPKs (Schweighofer et al. 2007) are responsible for conducting signals to the nucleus, where they phosphorylate transcription factors and activate hormonal cascades, such as those mediated by jasmonic acid (JA) and salicylic acid (SA), two key regulators of defense gene expression and defense-compound biosynthesis (Wu and Baldwin 2009). The second pathway is initiated when H_2_O_2_ activates ion channels, resulting in influxes of cytosolic Ca^2^⁺ that induce the activity of calcium-dependent protein kinases (CPKs) (Schweighofer et al. 2007; Iida et al. 2019; Yuan et al. 2021). In this case, CPKs are thought to phosphorylate RBOH proteins, reinforcing the generation of H_2_O_2_ and O_2_^−^ in the apoplast and amplifying the defense signal (Yuan et al. 2021) (Fig. 1).

## Conceptualizing plant stress in response to mite attacks

**Fig. 2:**
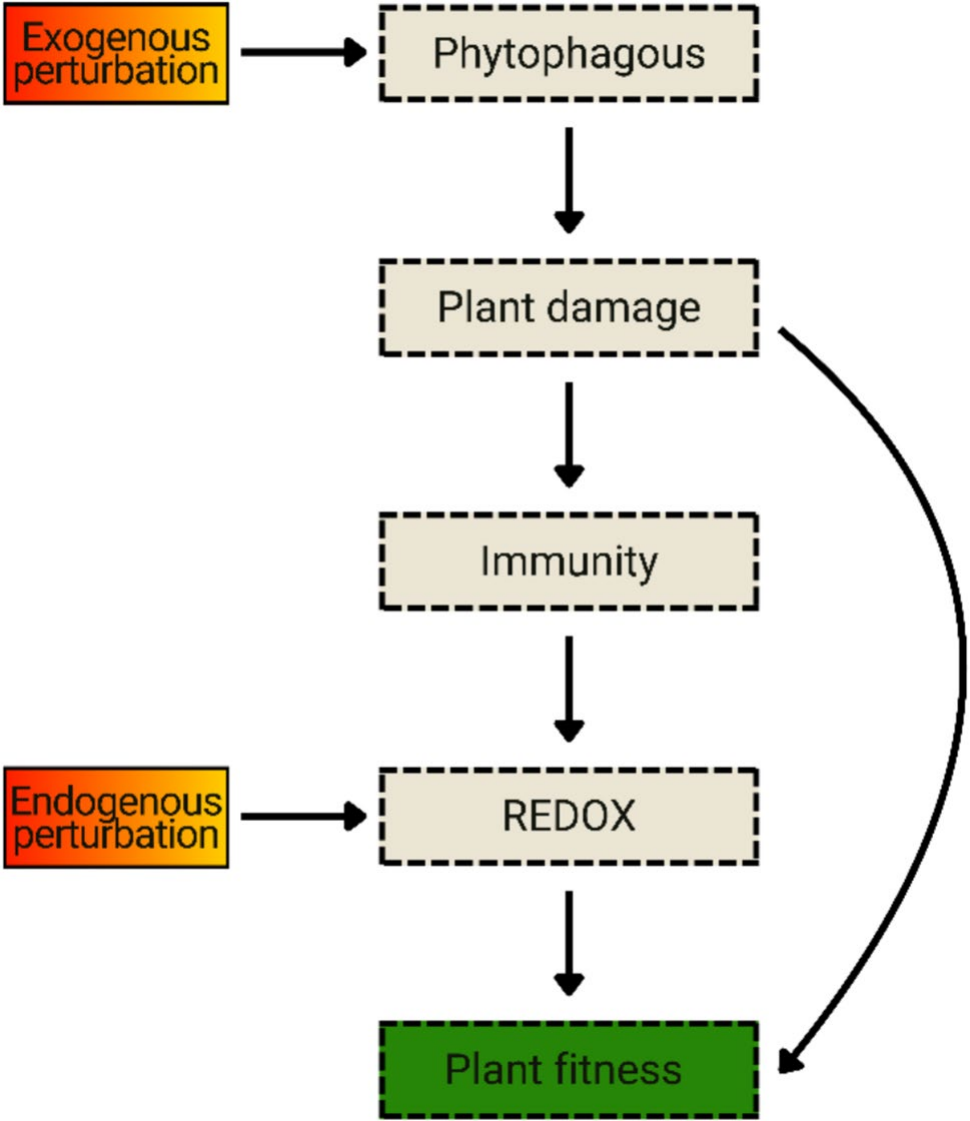
From herbivory to stress: a conceptual view of mite-induced stress. Phytophagous mites act as exogenous sources of disturbance to the plant. In response to their mechanical damage, the immune system triggers redox oscillations to signal the synthesis of defenses against herbivory. In this hypothetical case, where the plant is susceptible, the infestation becomes established over time, and these transient signaling influxes intensify, becoming a source of endogenous disturbances. In this scenario, both sources act simultaneously, manifesting a state of severe stress that is directly reflected in the plant’s physiological development and fitness

The concept of plant stress is based on parameters that indicate the partial or total breakdown of the plant’s physiological homeostasis, which is not a linear reaction to a given environmental perturbation (Lichtenthaler et al. 1996; Mosa et al. 2017). The assessment of plant stress states in response to abiotic factors is strongly based on oxidative parameters and their potential impacts on the physiological and reproductive development of plants (Schneider et al. 2019; Chauhan et al. 2023). Regarding plants under herbivore attack, Gogoi et al. (2024) compiled evidence suggesting that a stressful state is consistently associated with the combination of physical damage, transient redox disturbances linked to plant immunity, and their subsequent impacts on essential physiological traits. To illustrate, the soybean genotype most sensitive to infestation by the phytophagous mite *Tetranychus ludeni* exhibited a state of severe stress, characterized by the loss of photosynthetic pigments and senescent leaves, in response to intense oxidative damage and a high mite infestation (Wurlitzer et al. 2024b). In this sense, the stress induced by phytophagous mites apparently results from two sources that directly reflect on the plant’s fitness (Fig. 2) (Züst and Agrawal 2017).

## Predatory mites: allies in plant stress mitigation

The order of arrival is a factor in the interaction between pest and predator mites (Pérez-Hedo et al. 2022). Various species of predatory mites from the Phytoseiidae family have a generalist habit and can be on the host plant even before the presence of the phytophagous mite (Pappas et al. 2017; Pérez-Hedo et al. 2022). Thus, in this topic, we consider a scenario in which the plants are previously infested by phytophagous mites. We also disregard any type of predator mite behavior (specialist or generalist).

### Effect of predatory mites on plant redox maintenance

Antioxidant defenses play a key role in fine-tuning early immune system signals in plants under herbivory. We recently demonstrated that infestation by *Tetranychus ludeni* Zacher (Tetranychidae) triggered a transient oxidative burst in soybean plants, marked by reduced H_2_O_2_ accumulation due to peroxidase activity immediately after infestation, which characterizes a non-stressful transient redox state (Wurlitzer et al. 2025). In a mechanistic analogy, similar to the catalytic functions of these enzymes, several studies have reported the positive effects of predatory mite foraging on the suppression of phytophagous mites (Fontes and Valadares-Inglis 2020; Moraes et al. 2024). In our recent study on the functional responses of predatory mites in controlling the phytophagous mite *T. urticae*, *Phytoseiulus macropilis* Banks stood out for its faster attacks compared to *Neoseiulus californicus* (McGregor) (Phytoseiidae), although both species exhibited high control efficiency (Siebert et al. 2025). These results suggest that predatory mites, when released in the field as a management strategy, could act as allies of the antioxidant defenses of host plants (Peláez-Vico et al. 2024; Fontes and Valadares-Inglis 2020; Siebert et al. 2025).

On the other hand, based on this assumption, the absence of predatory mites favors infestation (Wurlitzer et al. 2024a, b). Therefore, the ROS signals generated early by the plant’s immune responses may become intensified (Gogoi et al. 2024), making fluctuations in redox homeostasis more difficult to compensate for solely by antioxidant defenses. In this case, the accumulation of H_2_O_2_, for example, in the presence of metal ions essential to plant metabolism, leads to the formation of the hydroxyl radical (•OH) (Smirnoff and Arnaud 2019). The increased concentration of •OH marks the transition from a controlled signaling state to endogenous oxidative stress, whose severity depends on the intensity and duration of the disturbance (Smirnoff and Arnaud 2019). The •OH culminates in lipid peroxidation (Fig. 1), one of the most destructive processes for plant cells (Wurlitzer et al. 2024a, b). During this event, the oxidation of proteins and membranes inactivates organelles, causing the cellular machinery to collapse (Smirnoff and Arnaud 2019). Ultimately, the late effects of this oxidative disturbance are manifested by the reduction of photosynthetic pigments, culminating in chlorosis that may directly compromise plant development (Chachar et al. 2025).

### Effect of predatory mites on maintaining plant fitness

The biological control of phytophagous mites through the release of predatory mites is well established and stands out as a sustainable alternative without the risk of resistance development (Fontes and Valadares-Inglis 2020). Among these natural enemies, mites of the family Phytoseiidae have shown remarkable potential and have been the target of studies for more than six decades across a wide range of crops (Fontes and Valadares-Inglis 2020; Moraes et al. 2024). However, knowledge about the benefits of mitigating plant physiological stress by suppressing phytophagous mite infestations is limited. To demonstrate this new perspective, we conducted a meta-analysis on the effect of predatory mites on physiological parameters that integrate plant fitness.

#### Literature search and inclusion criteria

To characterize plant fitness, a preliminary survey considered parameters associated with plant growth, photosynthesis, and reproduction, as proposed by Züst and Agrawal (2017). Based on the Preferred Reporting Items for Systematic Reviews and Meta-Analyses (PRISMA) (Moher et al. 2009; Schneider et al. 2025), we conducted searches in the Scopus and Google Scholar databases up to the 10th results page using the following keywords: *“(predatory mite) AND (biological control) AND (phytophagous mites) AND (number of fruits)”.* The term “*number of fruits”* was then replaced in independent searches for each of the following terms: *fruit weight, number of seeds, seed weight, chlorophyll content, number of leaves, leaf area,* and *relative height growth rate*. Additionally, the reference lists of the identified publications were consulted for other relevant articles. About 800 articles were identified, of which approximately 750 were excluded because they were review articles, theses, dissertations, or articles that evaluated only the effect of natural enemies on phytophagous control. The remaining studies were submitted to the screening stage based on the following criteria: (*i*) Experiments that specifically involved the interaction between plants, phytophagous mites, and predatory mites; (*ii*) Experiments that reported the effect of foraging predatory mites on at least one trait involving plant fitness; and (*iii*) Experiments that presented measures of dispersion.

#### Effect measures and meta‑analysis

After screening, nine articles were selected, resulting in 93 individual units of analysis (Supplementary Table S1) that presented the following characteristics or variables of interest: chlorophyll, chlorotic leaf, damaged leaves, fruit number, fruit weight, leaf area, leaf number, relative height growth rate (RHGR), stem damage, stem diameter, and yield. Data were extracted from the figures using Web Plot Digitizer (Burda et al. 2017). No distinction was made regarding experimental settings (laboratory, greenhouse, or field), plant species, mite species, predator release method (mass release or natural attraction), and the foraging duration, which explains the high levels of heterogeneity (Supplementary Table S2). To estimate the foraging effects of predatory mites on the traits of interest, we calculated the response ratio of the natural logarithm as a metric effect size (Cooper et al. 2019). The effect was calculated using the natural logarithm of the ratio between the considered variable of the treatment group (phytophagous mite + predator) and the control group (phytophagous mite). The meta-analysis was performed using a random-effects model, as heterogeneity was expected between studies. The effect measures were calculated using the escalc function of *metafor* (Viechtbauer 2010). A forest plot was created using ggplot2. Both the effect measures and the forest plot were created in the software RStudio version 3.6.1 (R Core Team 2019).

#### Publication bias

A funnel plot was used to assess publication bias (Terrin et al. 2003; Rosenberg 2005) and did not indicate evidence of bias (Supplementary Fig. S1) and showed symmetry between studies. Significant variability was observed between the studies (Higgins et al. 2003), with heterogeneity values reaching 100% (Supplementary Table S2). The meta-analysis indicated that predatory mite foraging positively influences plant fitness.

## Results

**Fig. 3:**
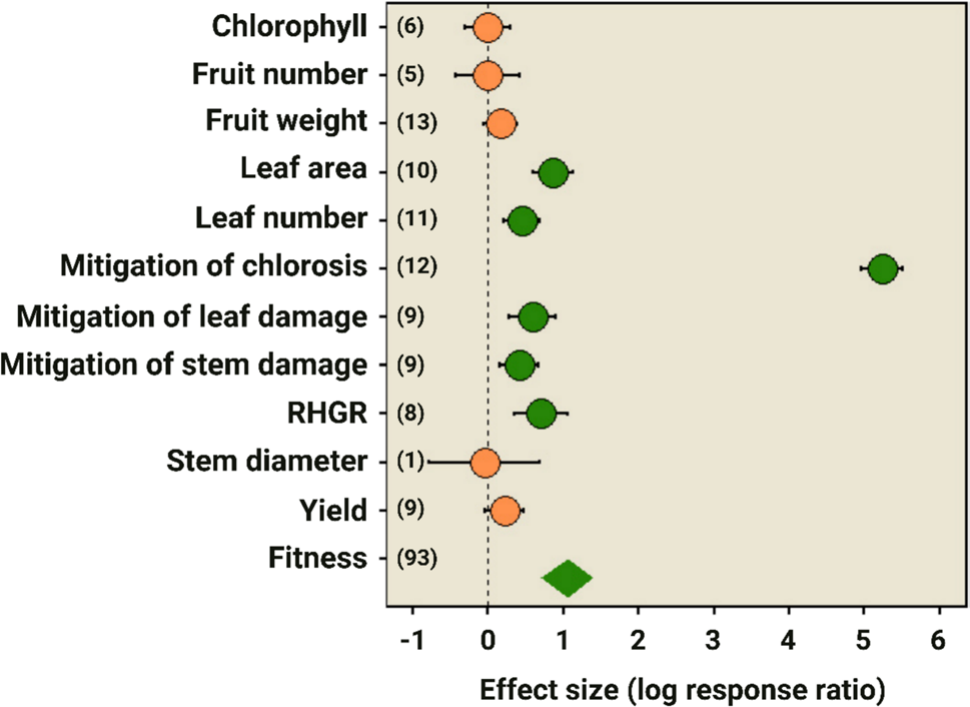
Forest plot showing the effect (log response ratio) and the confidence interval (95% CI) of the variables chlorophyll, fruit number, fruit weight, leaf area, leaf number, mitigation of chlorosis, mitigation of leaf damage, mitigation of stem damage, relative growth rate of plants (RHGR), stem diameter, harvest index, and overall plant fitness in response to foraging of predatory mites on phytophagous mites. Sample sizes are given in parentheses. The orange circles represent a neutral effect, and the green circles represent a positive effect of the variables. The green diamond indicates a positive effect on the overall fitness

Compared to the group of plants infested only by phytophagous mites, the fitness of the plants benefited from the foraging of predatory mites (1.054; 95% CI: 0.709 to 1.398; *p* ≤ 0.001). The main indices that benefited were leaf area (0.858; 95% CI: 0.591 to 1.124; *p* ≤ 0.000), number of leaves (0.444; 95% CI: 0.212 to 0.676; *p* ≤ 0.000), and RHGR (0.695; 95% CI: 0.341 to 1.048; *p* ≤ 0.000), in addition to mitigation of the worsening of foliar chlorosis (5.237; 95% CI: 4.965 to 5.510; *p* ≤ 0.000), leaf damage (0.587; 95% CI: 0.284 to 0.890; *p* ≤ 0.000), and stem damage (0.409; 95% CI: 0.149 to 0.670; *p* = 0.002). Finally, although the effects involving chlorophyll (− 0.009; 95% CI: − 0.307 to 0.289; *p* = 0.952), fruit number (− 0.008; 95% CI: − 0.439 to 0.424; *p* = 0.972), fruit weight (0.165; 95% CI: − 0.059 to 0.389; *p* = 0.148), stem diameter (− 0.051; 95% CI: − 0.782 to 0.681; *p* = 0.892), and productivity (0.214; 95% CI: -0.036 to 0.464; *p* = 0.092) were not statistically significant (Fig. 3), these results did not indicate negative effects. Therefore, although these results do not constitute proof of the absence of an effect, they may suggest a neutral effect, especially considering the width of the confidence intervals.

## Discussion

In agroecosystems, biological agents have been employed to reduce the metabolic costs associated with plant defense against herbivores, pathogens, and endogenous oxidative stress (Ku et al. 2024). A notable example is the introduction of the bacterium *Paraburkholderia* sp. GD17 in tomato plants (*Solanum lycopersicum* L.) infected by the fungus *Botrytis cinerea* Pers.:Fr. In addition to reducing the severity of the disease, it acted synergistically with the plant’s oxidative defenses, contributing to maintaining the plant’s fitness (Gu et al. 2023). In this context, although mites are distinct from microorganisms, the late infestation by *T. ludeni*, for example, in soybean plants induces severe oxidative stress, cell death, and a loss of photosynthetic capacity, leading to chlorotic and senescent leaves (Wurlitzer et al. 2024b). As a safe management strategy, considering the data from this meta-analysis and those of Gu et al. (2023), foraging by predatory mites could mitigate this perturbation, thus decreasing leaf chlorosis in plants.

Supporting this proposal, our unpublished data revealed that Phytoseiidae predatory mites efficiently suppress *Tetranychus* sp. infestation in soybean plants, preserving approximately 20% of the maximum quantum yield of photosystem II (PSII) (Fv/Fm) and 60% of the photochemical yield of PSII (ΦPSII). This evidence suggests that the redox disturbances resulting from the plant’s immune responses do not intensify with the foraging of the predatory mite. PSII reaction centers are dependent on the integrity of the D1 protein for their functionality, which is sensitive to the action of H_2_O_2_ and O_2_^−^ at elevated levels (Nishiyama et al. 2011). Thus, if intense oxidative events occurred, the degradation of these structures and essential proteins would be marked by such photosynthetic parameters (Sahu et al. 2022; Gogoi et al. 2024). In cucumber plants, for example, the application of the pesticide chlorpyrifos (often used for insect and acaricide control) triggered severe physiological stress events, marked especially by a reduction in Fv/Fm by more than 10% and ΦPSII by approximately 30% (Xia et al. 2006). Together, this evidence suggests that predatory mites are efficient mitigating agents of physiological oxidative disturbances involved in the photosystem apparatus.

**Fig. 4:**
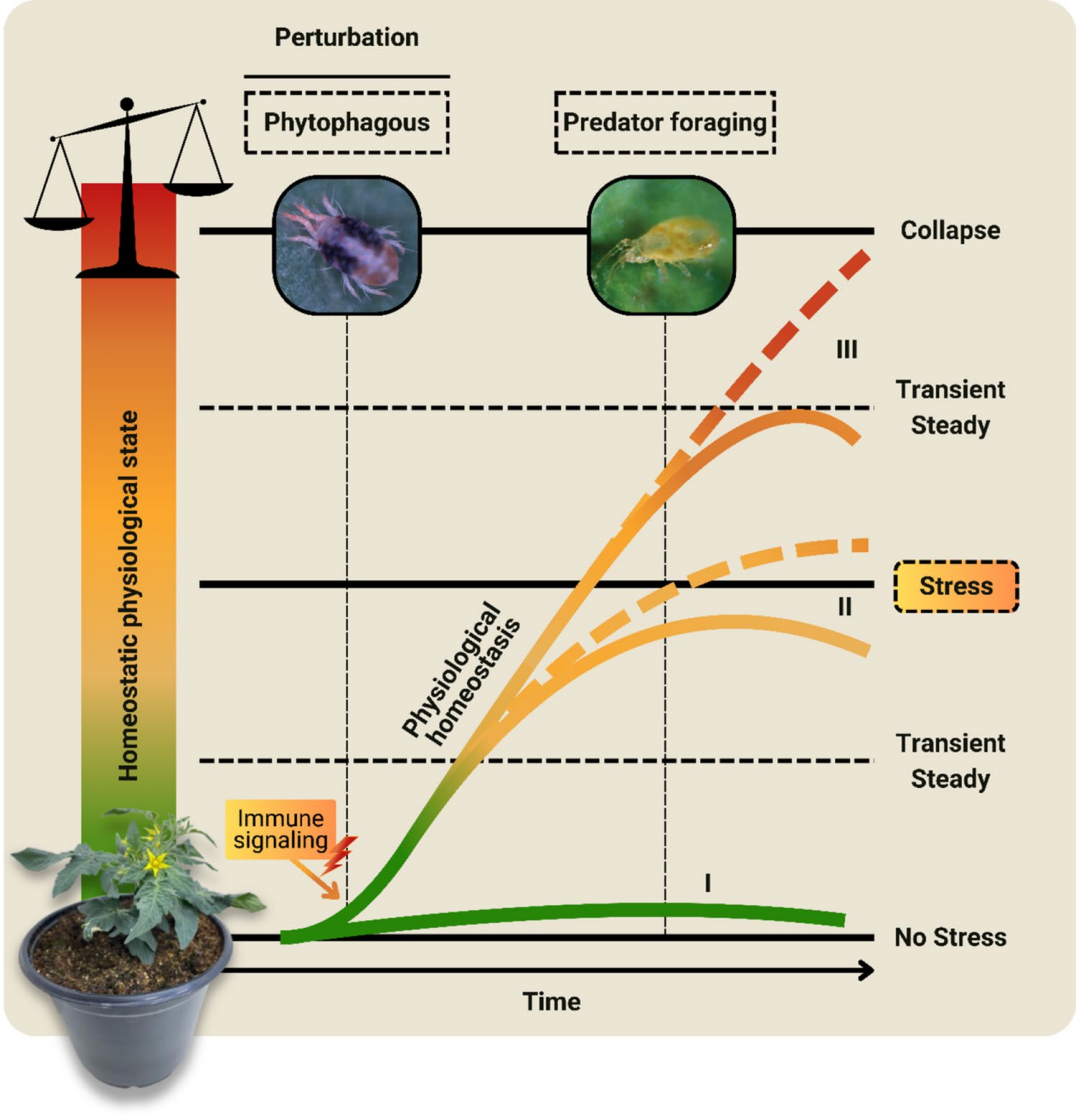
Hypothetical schematic model of the benefits of predatory mite foraging in mitigating plant physiological stress. When attacked by phytophagous mites, plants accumulate reactive oxygen species (ROS) to signal defenses. This process is related to fluctuations in redox homeostasis, adjusted by antioxidant defense systems (balance). As the infestation becomes established, the intensity of the disturbance increases, resulting in endogenous oxidative disturbances that alter the state of the plant’s physiological homeostasis (greenish extremity = normal state; yellow middle region = stress; reddish extremity = collapse). Plants without infestations (I) have only normal or essential redox oscillations, characterizing a non-stressed physiological state. Infested plants with more robust metabolic efficiency (II—dotted line) may experience reversible physiological stress; however, with the foraging of predatory mites, this state tends to be mitigated (II—continuous line). Plants with lower metabolic efficiency (III—dotted line), in the absence of predatory mites, can present the beginning of the collapse of their leaves or even the complete death of their parts; however, with the presence of natural enemies (III—continuous line), this physiological homeostasis can reach a transitory state between stress and collapse. The proportions of the graphic elements are illustrative only. The scheme disregards the morphological characteristics of the plants and behavioral characteristics of predatory mites and phytophagous mites and assumes an equivalent hypothetical population density for both plants. Phytophagous mite represented by *Tetranychus urticae*. Predatory mite represented by *Neoseiulus californicus*. Plant represented by *Solanum lycopersicum*

Assuming that the primary role of the leaves is to convert sunlight into sucrose for storage, especially in the fruit (Keller et al. 2021), mitigating the progression of leaf damage by predatory mites contributes to new photosynthetically active tissues that ensure a functional leaf apparatus. Considering that metabolic responses in plants oscillate in a spatiotemporal dynamic manner (Galviz et al. 2022), it is plausible that traits associated with reproduction and stem diameter require longer periods to exhibit measurable responses, which may explain the absence of effects observed in our analysis. Although components like these also depend significantly on the metabolic capacity of each species or even genotype (Silveira and Sousa 2024). In summary, because of the mitigation of endogenous oxidative disturbances, a non-stressful physiological state may result from foraging by natural enemies, as indicated by the maintenance of plant fitness (Fig. 4).

## Attracting predatory mites: a stress-mitigating strategy?

Here, we propose a conceptual model in which initial oxidative disturbances in infested plants contribute to the synthesis of volatile organic compounds (VOCs), which recruit predatory mites.

**Fig. 5:**
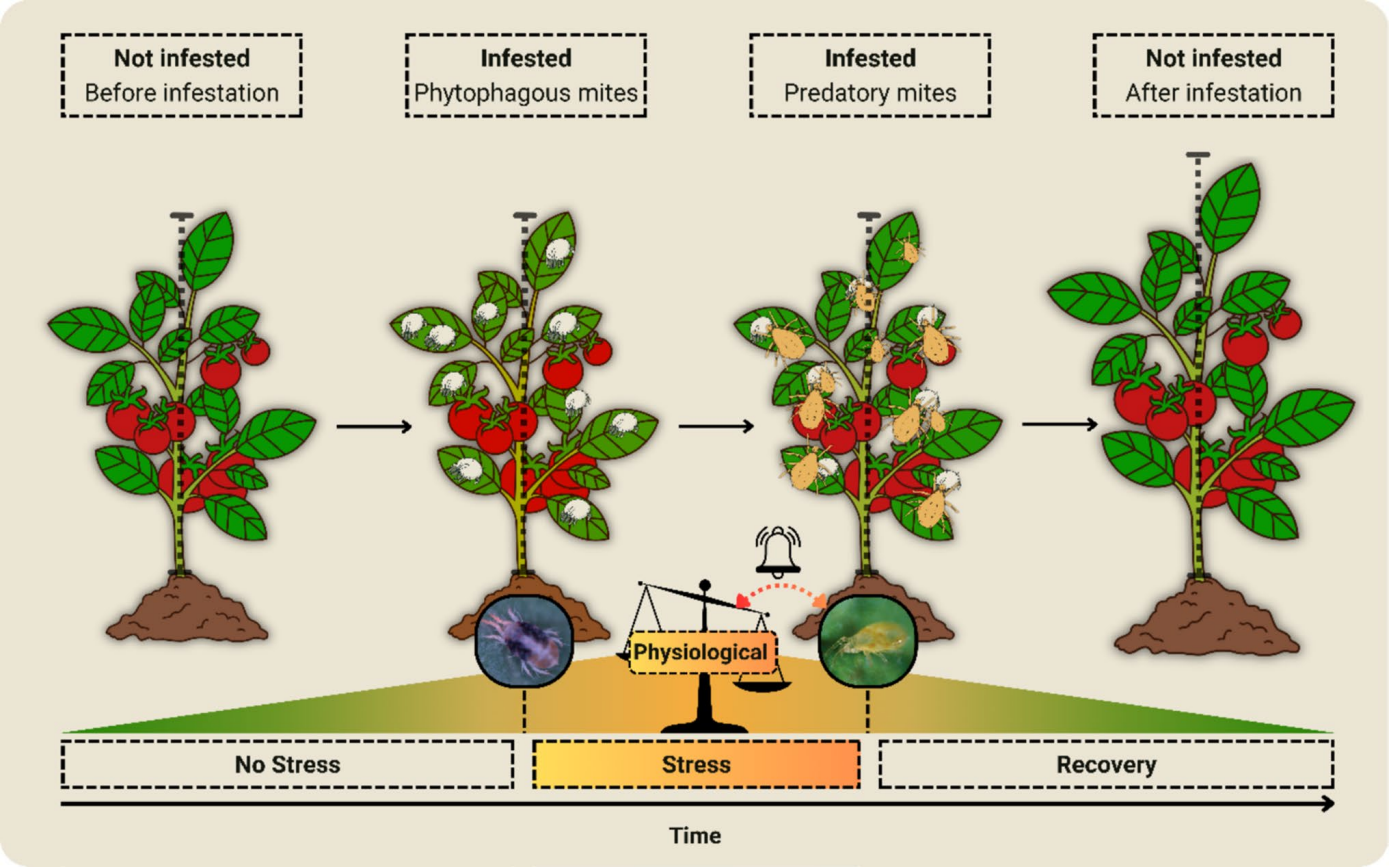
Hypothetical schematic model of how the physiological state of plant stress contributes to the attraction of predatory mites. When attacked by phytophagous mites, non-stressed plants (greenish extremity on the left) proceed from their basal physiological state to a condition of stress (yellow intermediate region). When reaching this threshold state, the oxidative endogenous disturbance is not supported by antioxidant defenses, contributing to the emission of volatiles, which attract predatory mites (alert represented by the bell). Predatory mites, by suppressing phytophagous infestation over time, initiate the recovery of the essential physiological state and maintain plant fitness (greenish extremity on the right). The scheme disregards the morphological characteristics of plants and behavioral characteristics of predatory and phytophagous mites. The proportions of the graphic elements are illustrative only. Phytophagous mite represented by *Tetranychus urticae*. Predatory mite represented by *Neoseiulus californicus*. Plant represented by *Solanum lycopersicum*

Infestation by *T. urticae*, for example, upregulated genes involved in the biosynthesis of terpenoids and phenolic compounds in tomato plants on the first day of infestation; however, the attraction of the predatory mite *Phytoseiulus persimilis* Athias-Henriot (Phytoseiidae) was recorded only on the fourth day, when homoterpene (*E,E*)-4,8,12-trimethyltridec-1,3,7,11-tetraene (TMTT) and methyl salicylate (MeSA) were emitted (Kant et al. 2004). Niinemets et al. (2013) describe a similar mechanism, highlighting that this immune system is dependent on basal ROS signals, which function as necessary alarms for the activation of specific genes involved in the synthesis and emission of VOCs. Building on these observations, we hypothesize that the delayed recruitment of predatory mites is temporally regulated and occurs when oxidative disturbances exceed a still unknown physiological threshold (Fig. 5). Thus, continuous herbivory by mites on plants may trigger an alarming ROS state, which induces the emission of VOCs to recruit natural enemies and mitigate an intense state of stress.

We can also propose a mechanism by which plants could indirectly mitigate the stress of neighboring plants attacked by phytophagous mites. In addition to VOCs, plants may also present specific characteristics that serve as secondary nutritional resources for natural enemies (Pearse et al. 2020). Conservative biological control uses this basis to establish pest management strategies in agroecosystems (Fontes and Valadares-Inglis 2020). For example, the presence of pollen in sunn hemp (*Crotalaria juncea* L.) plants has been shown to function as a secondary food resource, supporting the biological maintenance of phytoseiid mites in coffee orchards, which efficiently suppress Tetranychidae phytophagous mite populations (Consolação Rosado et al. 2021). These reservoir plants of natural enemies could help mitigate physiological disturbances caused by phytophagous mites in their neighboring plants, potentially serving as a form of indirect defense to mitigate severe disturbances.

## Conclusions and perspectives

The results presented here provide clear evidence that foraging by predatory mites on phytophagous mites contributes to mitigating plant damage. The meta-analysis provides evidence that these natural enemies act indirectly, reducing the pressure of these herbivores, which limits leaf and stem damage, and contributes to a better physiological performance of the host plant, marked by the mitigation of chlorosis, loss of area, and decreased number of leaves.

Therefore, our results suggest a new approach to predatory mites that goes beyond biological control, supporting the concept that predatory mites can be efficient in sustainable production, not only because of the efficient biological management of phytophagous mites, reduction of chemical residues in plants, and lower environmental impacts, but also because they reduce the metabolic costs of the plants. The complexity of evaluating these concepts both in the laboratory and in the field is one of the great limitations for the development of studies with such a focus. Given that the impacts of phytophagous mites are directly related to the reduction in photosynthetic performance, we propose a set of rapid and efficient parameters that can assess the intensity of plant stress and, therefore, the effect of predatory mites. To verify these benefits, a comparison between management techniques (biological and chemical) in future experiments is necessary.

We recommend that researchers in the field of biological control include evaluations of physiological measurements such as chlorophyll *a*, *b*, and total chlorophyll content, efficiency of photosystem II (Fv/Fm), instantaneous chlorophyll fluorescence (Ft), stomatal conductance, and membrane integrity based on the electrical conductivity of water (Gu et al. 2023; Schneider et al. 2023). These measurements should be performed during the attack by phytophagous mites (i.e., before the release of predatory mites) and after the introduction of natural enemies. To strengthen these analyses, it will be essential to consider biochemical indicators involved in redox metabolism, such as ROS, lipid peroxidation, and antioxidant defenses. Based on these measures, it will be possible to refine management techniques, for example, the selection of more efficient agents to promote faster physiological recovery, even before plants reach a critical physiological threshold.

## Supplementary Information

Below is the link to the electronic supplementary material.

Supplementary file1 (PDF 152 KB)

Supplementary file2 (PDF 312 KB)

Supplementary file3 (PDF 122 KB)

## Data Availability

Data will be available upon request.

## Abbreviations

^1^O_2_: Singlet oxygen
CPKs: Calcium-dependent protein kinases
DAMPs: Damage-associated molecular patterns
EAMPs: Egg-associated molecular patterns
H_2_O_2_: Hydrogen peroxide
HAMPs: Herbivore-associated molecular patterns
JA: Jasmonic acid
MAPKs: Mitogen-activated protein kinase
O_2_.^.^^−^: Superoxide
OH^.^: Hydroxyl radical
PRRs: Pattern recognition receptors
RBOHs: Respiratory burst oxidase homologs
RHGR: Relative height growth rate
ROS: Reactive oxygen species
SA: Salicylic acid
