# Supplementary material for: Can biological control involving predatory mites mitigate plant stress caused by phytophagous mites?
Source: Planta. 2026 Apr 29;263(6):143. doi: 10.1007/s00425-026-05004-z (PMC13124832; doi:10.1007/s00425-026-05004-z)
Supplement: Supplementary file 1 — Supplementary file1 (PDF 152 KB) [file 425_2026_5004_MOESM1_ESM.pdf]

Can biological control involving predatory mites mitigate plant stress caused by phytophagous mites?

Wesley Borges Wurlitzer[a,b,1,*], Julia Renata Schneider[a,b,c,1], Mateusz Labudda[d], Julia Huppes Majolo[a,c], Marcelo Lattarulo Campos[e,h], Joaquim A. G. Silveira[f,h], Daniel Guimarães Silva Paulo[c,g,h], Maria Goreti de Almeida Oliveira[c,h], Noeli Juarez Ferla[a,b,i,h]

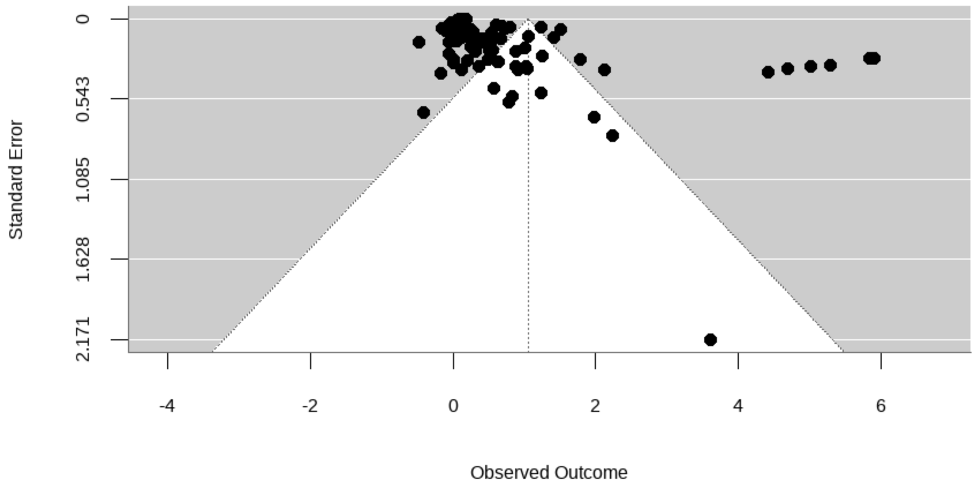

Fig. S1 Funnel plot to explore publication bias on variables related to plant fitness in response to foraging by predatory mites
