# Supplementary material for: Can biological control involving predatory mites mitigate plant stress caused by phytophagous mites?
Source: Planta. 2026 Apr 29;263(6):143. doi: 10.1007/s00425-026-05004-z (PMC13124832; doi:10.1007/s00425-026-05004-z)
Supplement: Supplementary file 2 — Supplementary file2 (PDF 312 KB) [file 425_2026_5004_MOESM2_ESM.pdf]

Can biological control involving predatory mites mitigate plant stress caused by phytophagous mites?

Wesley Borges Wurlitzer[a,b,1,*], Julia Renata Schneider[a,b,c,1], Mateusz Labudda[d], Julia Huppes Majolo[a,c], Marcelo Lattarulo Campos[e,h], Joaquim A. G. Silveira[f,h], Daniel Guimarães Silva Paulo[c,g], Maria Goreti de Almeida Oliveira[c,h], Noeli Juarez Ferla[a,b,i,h]

Table S1 Studies included in this meta-analysis

**Ref.[1]**	**Moderators**	**IUA[2]**	**Phyt.[3]**	**Phyt. + Pred.[4]**	**SD Phyt.[5]**	**SD Phyt. + Pred.[6]**	**Rep. Phyt.[7]**	**Rep. Phyt. + Pred. [8]**	**Plant species**	**Phytophagous species**	**Predator species**
Elhalawany et al. (2024)	Yield	1	546.20	593.40	1.88	0.94	20	5	*Psidium guajava* L.	*Tegolophus guavae* (Boczek) *Brevipalpus phoenicis* (Geijskes) *Tetranychus urticae* Koch	*Neoseiulus californicus* (McGregor)
		2	546.20	621.20	1.88	0.76	20	5			
		3	546.20	649.90	1.88	0.72	20	5			
		4	548.40	599.30	2.10	0.92	20	5			
		5	548.40	631.10	2.10	0.74	20	5			
		6	548.40	655.50	2.10	0.65	20	5			
Rodríguez-Cruz et al. (2017)	Fruit number	7	3.31	7.22	2.55	7.22	5	5	*Capsicum frutescens* L.	*Polyphagotarsonemus latus* (Banks)	*Amblyseius herbicolus* (Chant)
		8	3.31	7.63	2.55	6.86	5	5			*Neoseiulus barkeri* Hughes
	Fruit weight	9	0.10	0.19	0.04	0.09	5	5			*A. herbicolus*
		10	0.10	0.25	0.04	0.16	5	5			*N. barkeri*
Hartvigsen et al. (1995)	Leaf number	11	16.84	16.84	7.22	7.22	4	4	*Populus deltoides* Marsh.	*T. urticae*	*Phytoseiulus persimilis* Athias-Henriot
		12	11.13	15.34	4.51	2.42	4	4			
		13	8.99	13.52	2.79	0.87	4	4			
		14	7.95	17.69	0.87	0.87	4	4			
		15	10.38	17.87	0.87	2.96	4	4			
		16	16.48	16.48	1.04	1.04	4	4			
		17	10.91	18.91	4.52	1.39	4	4			
		18	10.04	21.00	1.22	0.52	4	4			
		19	8.12	16.13	0.52	1.22	4	4			
		20	9.51	17.52	0.70	0.70	4	4			
	Leaf area	21	990.00	990.00	381.00	381.00	4	4			
		22	609.50	780.90	95.20	266.70	4	4			
		23	298.06	838.18	176.37	275.58	4	4			
		24	264.99	1190.92	33.07	88.18	4	4			
		25	430.33	1488.50	44.10	66.20	4	4			
		26	959.40	959.40	385.80	385.80	4	4			
		27	705.90	1257.05	639.32	330.69	4	4			
		28	656.30	1271.75	128.61	229.65	4	4			
		29	298.06	1225.82	27.56	284.76	4	4			
		30	343.99	1207.45	165.34	174.53	4	4			
	Relative height growth rate	31	0.06	0.04	0.07	0.02	4	4			
		32	0.08	0.13	0.04	0.03	4	4			
		33	0.05	0.12	0.01	0.05	4	4			
		34	0.01	0.10	0.00	0.06	4	4			
		35	0.06	0.10	0.01	0.04	4	4			
		36	0.16	0.15	0.07	0.03	4	4			
		37	0.05	0.12	0.03	0.03	4	4			
		38	0.00	0.11	0.01	0.03	4	4			
Marcossi et al. (2025)	Fruit number	39	4.61	4.36	1.45	0.63	5	5	*Solanum lycopersicum* L.	*Aculops lycopersici* (Massee, 1937)	*A. herbicolus*
		40	4.61	2.88	1.45	0.47	5	5			*Homeopronematus anconai* (Baker)

	Fruit weight	41	29.69	33.40	17.98	15.90	5	5			*A. herbicolus*
		42	29.69	42.99	17.98	17.28	5	5			*H. anconai*
Wang et al. (2025)	Chlorophyll	43	61.35	64.04	3.26	3.84	3	3	*Fragaria × ananassa* Duch.	*T. urticae*	*N. californicus*
		44	61.35	58.27	3.26	3.27	3	3			*P. persimilis*
		45	61.35	63.27	3.26	2.31	3	3			*N. californicus* + *P. persimilis*
		46	51.03	50.39	2.36	1.46	3	3			*N. californicus*
		47	51.03	49.84	2.36	0.91	3	3			*P. persimilis*
		48	51.03	49.03	2.36	1.72	3	3			*N. californicus* + *P. persimilis*
	Fruit weight	49	22.8	19.60	1.20	1.80	3	3			*N. californicus*
		50	22.8	20.00	1.20	1.99	3	3			*P. persimilis*
		51	22.8	25.99	1.20	1.01	3	3			*N. californicus* + *P. persimilis*
		52	25.86	26.09	3.20	2.06	3	3			*N. californicus*
		53	25.86	33.17	3.20	1.37	3	3			*P.persimilis*
		54	25.86	30.09	3.20	2.40	3	3			*N. californicus* + *P. persimilis*
Mena et al. (2020)	Leaf number	55	30.9	32.90	0.52	0.52	3	3	*Carica papaya* L.	*T. urticae*	*P. persimilis*
	Stem diameter	56	22.3	21.20	1.39	1.21	3	3			
	Fruit number	57	3.2	2.70	1.21	1.39	3	3			
	Fruit weight	58	0.9	1.10	0.35	0.35	3	3			
Argolo et al. (2020)	Mitigation of Chlorosis	59	0.83	0	0.81	0.00	15	15	*Citrus limon* L.	*Brevipalpus yothersi* Baker	*Amblyseius largoensis* (Muma)
		60	1.08	0	0.89	0.00	15	15			
		61	1.49	0	1.08	0.00	15	15			
		62	1.97	0	1.36	0.00	15	15			
		63	3.39	0	0.74	0.00	15	15			
		64	3.67	0	0.85	0.00	15	15			
		65	0.83	0	0.81	0.00	15	15			*A. largoensis* + *Hemicheyletia bakeri* (Ehara)
		66	1.08	0	0.89	0.00	15	15			
		67	1.49	0	1.08	0.00	15	15			
		68	1.97	0	1.36	0.00	15	15			
		69	3.39	0	0.74	0.00	15	15			
		70	3.67	0	0.85	0.00	15	15			
Patenaude et al. (2020)	Yield	71	280.69	523.45	130.07	278.73	6	6	*F. × ananassa*	*Phytonemus pallidus* (Banks)	*Neoseiulus cucumeris* (Oud.)
		72	652.41	864.83	111.50	111.48	6	6			
		73	933.1	1403.45	241.57	278.73	6	6			
	Fruit weight	74	7.29	8.21	2.06	1.20	6	6			
		75	2.9	3.21	0.47	0.73	6	6			
Pijnakker et al. (2022)	Mitigation of leaf damage	76	53.5	50.21	4.99	12.11	3	3	*S. lycopersicum*	*A. lycopersici*	*Phytoseiulus macropilis* (Banks)
		77	53.5	7.41	7.05	12.10	6	6			*N. californicus*
		78	53.5	9.05	7.05	6.05	6	6			*Amblyseius andersoni* (Chant)
		79	53.5	5.76	7.05	11.10	6	6			*Neoseiulus fallacis* (Garman)
		80	53.5	15.64	7.05	19.16	6	6			*Galendromus occidentalis* (Nesbitt)
		81	53.5	30.45	7.05	10.09	6	6			*Amblyseius swirskii* Athias-Henriot
		82	53.5	48.15	7.05	12.10	6	6			*Amblydromalus limonicus* (Garman & McGregor)
		83	53.5	51.03	4.99	12.11	3	3			*Euseius ovalis* (Evans) + polen

		84	53.5	39.51	7.05	16.12	6	6			*A. swirskii* + polen
	Mitigation of stem damage	85	79.35	29.15	14.87	12.88	6	6			*N. fallacis*
		86	79.35	28.34	14.87	21.82	6	6			*N. californicus*
		87	79.35	27.53	14.87	5.95	6	6			*A. andersoni*
		88	79.35	47.37	14.87	11.90	6	6			*G. occidentalis*
		89	79.35	56.68	14.87	13.89	6	6			*A. swirskii*
		90	79.35	61.54	14.87	17.86	6	6			*A. swirskii* + polen
		91	79.35	83.4	14.87	11.27	6	6			*A. limonicus*
		92	79.35	78.14	10.51	4.21	3	3			*P. macropilis*
		93	79.35	88.26	10.51	4.21	3	3			*E. ovalis* + polen

Description of the columns: **Ref.**[1] References; **IUA**[2] Individual units of analysis; **Phyt.**[3] Average of phytophagous mites; **Phyt. + Pred.**[4] Average of phytophagous mites + predatory mites; **SD Phyt.**[5] Standard deviation of phytophagous mites; **SD Phyt. + Pred.**[6] Standard deviation of phytophagous mites + predatory mites; **Rep. Phyt.**[7] Number of replicates with phytophagous mites; **Rep. Phyt. + Pred.**[8] Number of replicates with phytophagous mites + predatory mites.

References

Argolo PS, Revynthi AM, Canon MA, et al (2020) Potential of predatory mites for biological control of *Brevipalpus yothersi* (Acari: Tenuipalpidae). Biological Control 149: 104330. https://doi.org/10.1016/j.biocontrol.2020.104330

Elhalawany AS, Sanad AS, Kassem EMK (2024) Efficiency of *Neoseiulus californicus* (McGregor) (Acari: Phytoseiidae) for controlling three plant-feeding mites on guava trees in Egypt. Int J Acarol 50:714–720. https://doi.org/10.1080/01647954.2024.2404134

Hartvigsen G, Wait DA, Coleman JS (1995) Tri-Trophic Interactions Influenced by Resource Availability: Predator Effects on Plant Performance Depend on Plant Resources.

Marcossi Í, Francesco LS, Fonseca MM, et al (2025) Predatory mites as potential biological control agents for tomato russet mite and powdery mildew on tomato. J Pest Sci (2004) 98:251–263. https://doi.org/10.1007/s10340-024-01802-0

Mena YM, Mesa NC, Escobar A, Pérez S (2020) Evaluation of phytoseiidae mites and *Chrysoperla carnea* (Stephens) on the control *of Tetranychus urticae* in *Carica papaya* l. Agron Colomb 38:3–11. https://doi.org/10.15446/agron.colomb.v38n1.73271

Patenaude S, Tellier S, Fournier V (2020) Cyclamen mite (Acari: Tarsonemidae) monitoring in eastern Canada strawberry (Rosaceae) fields and its potential control by the predatory mite *Neoseiulus cucumeris* (Acari: Phytoseiidae). Canadian Entomologist 152:249–260. https://doi.org/10.4039/tce.2019.75

Pijnakker J, Hürriyet A, Petit C, et al (2022) Evaluation of Phytoseiid and Iolinid Mites for Biological Control of the Tomato Russet Mite *Aculops lycopersici* (Acari: Eriophyidae). Insects 13: 13121146. https://doi.org/10.3390/insects13121146

Rodríguez-Cruz FA, Janssen A, Pallini A, et al (2017) Two predatory mite species as potential control agents of broad mites. BioControl 62:505–513. https://doi.org/10.1007/s10526-017-9813-0

Wang E, Hou Z, Yan H, et al (2025) Combining two predatory mite species (Phytoseiidae) to control two-spotted spider mites in greenhouse strawberry production. Syst Appl Acarol. https://doi.org/10.11158/saa.30.1.9
