# Supplementary material for: Can biological control involving predatory mites mitigate plant stress caused by phytophagous mites?
Source: Planta. 2026 Apr 29;263(6):143. doi: 10.1007/s00425-026-05004-z (PMC13124832; doi:10.1007/s00425-026-05004-z)
Supplement: Supplementary file 3 — Supplementary file3 (PDF 122 KB) [file 425_2026_5004_MOESM3_ESM.pdf]

Can biological control involving predatory mites mitigate plant stress caused by phytophagous mites?

Wesley Borges Wurlitzer[a,b,1,*], Julia Renata Schneider[a,b,c,1], Mateusz Labudda[d], Julia Huppes Majolo[a,c], Marcelo Lattarulo Campos[e,h], Joaquim A. G. Silveira[f,h], Daniel Guimarães Silva Paulo[c,g], Maria Goreti de Almeida Oliveira[c,h], Noeli Juarez Ferla[a,b,i,h]

Table S2 Results of the heterogeneity analysis of plant fitness in response to foraging of predatory mites

Number of studies	Test for Heterogeneity				tau^2	SE	Tau	I^2	H^2
	Q	df	P	Significance					
93	13,573.48	92	$P \leq 0.001$	***	2.794	0.423	1.671	100.00%	15,5432.11

Q Q-statistic/Cochrane's Q (heterogeneity test); ***df*** degrees of freedom; ***P*** probability; ***SE*** effect size; **tau^2** tau, **I^2**, **H^2** (heterogeneity test).
